# Targeting phosphoinositide 3-kinases and histone deacetylases in multiple myeloma

**DOI:** 10.1186/s40164-021-00213-6

**Published:** 2021-03-04

**Authors:** Seiichi Okabe, Yuko Tanaka, Akihiko Gotoh

**Affiliations:** Department of Hematology, Tokyo Medical University, 6-7-1 Nishi-shinjuku, Shinjuku-ku, Tokyo, 160-0023 Japan

**Keywords:** Multiple myeloma, Histone deacetylase, phosphatidylinositol-3 kinase, Proteasome inhibitor

## Abstract

**Background:**

Multiple myeloma (MM) is a type of hematological malignancy affecting the functions of plasma cells. The treatment of MM patients has changed dramatically with the use of new agents. However, unfortunately, it is still incurable. Therefore, a new approach for treating MM is still needed to improve patient outcomes.

**Methods:**

Because the histone deacetylase (HDAC) and phosphoinositide 3-kinase (PI3K) pathway is a key signal in cancer cell biology, we investigated whether dual HDAC and PI3K inhibitors could suppress the myeloma cells.

**Results:**

Gene expression of HDACs is high in myeloma cells. CUDC-907, a dual inhibitor of PI3K and HDAC, inhibits HDAC activity. Akt activity and expression of BCL-XL, MCL-1, and NF-κB p65 were reduced by CUDC-907 in a dose-dependent manner. The number of apoptotic and caspase 3/7-positive cells also increased in the myeloma cells. Combined treatment of myeloma cells with carfilzomib and CUDC-907 increased cytotoxicity compared to that observed with each drug alone.

**Conclusions:**

Data from this study suggested that the administration of CUDC-907 might be a powerful strategy against myeloma cells, to enhance the cytotoxic effects of proteasome inhibitors.

## Background

Multiple myeloma (MM) is a B-cell malignancy that is characterized by the accumulation of clonal plasma cells in the bone marrow [1]. The majority of myeloma patients exhibit many complications. These include hypercalcemia, renal insufficiency, anemia, and bone lesions also known as CRAB symptoms [2]. All these complications require treatment. Over the past decade, one of the major advances in the management of patients who were diagnosed with symptomatic multiple myeloma has been the introduction of new drugs such as proteasome inhibitors (e.g., bortezomib and carfilzomib), immunomodulators (thalidomide and lenalidomide), and antibody therapies (SLAMF7 and CD38) as an addition to the existing therapeutic strategies. These have been used in combination with the existing therapy in clinical treatment [3, 4]. These new therapies prolong the progression-free survival and overall survival of patients with MM. Despite these improvements, MM remains an incurable condition for many patients because most patients become refractory during treatment [5]. Therefore, new strategies are still needed to increase the survival of MM patients.

Epigenetic changes, including histone deacetylation, may cause alterations that affect gene expression [6]. Aberrant expression of classical HDACs has been linked to a variety of tumors. Since HDACs play an important role in tumorigenesis, HDAC inhibitors represent a class of agents that have exhibited promising therapy for several cancers [7]. Moreover, class I HDACs play a significant role in the regulation of cell proliferation and cancer [7]. Recently, panobinostat, a pan-HDAC inhibitor, is clinically available in combination with bortezomib and dexamethasone for MM treatment [8].

Phosphatidylinositol-3-kinase (PI3K)/Akt and the mammalian target of rapamycin (mTOR) signaling pathways control multiple cellular processes, including metabolism and proliferation [9]. PI3K/Akt/mTOR signaling is one of the most important intracellular pathways, which plays a role in cell proliferation, and it is a frequently activated pathway in cancer. In particular, idelalisib, a PI3Kδ-specific inhibitor, has been approved by the United States Food and Drug Administration (FDA) and has been proven effective in the treatment of cancer, including relapsed indolent chronic lymphoid leukemia [10]. PI3K/Akt/mTOR is also aberrantly activated in a large proportion of MM patients [11]. Therefore, inhibiting the constitutively active PI3K pathway may become a new strategy for targeted cancer therapy.

Thus, HDAC and PI3K inhibition may be effective in myeloma cells. Since the inhibition of HDAC and/or PI3K may become a new strategy for targeted cancer therapy, we hypothesized that dual inhibition of HDAC and PI3K can reduce the growth of multiple myeloma. Therefore, we investigated whether the dual inhibition of HDAC and PI3K affects myeloma cells and reduces their growth in the presence of proteasome inhibitors by using MM cell lines.

## Methods

### Reagents

CUDC-907, pictilisib, and vorinostat were purchased from Selleck Chemicals (Houston, TX, USA). Carfilzomib was purchased from MedKoo Biosciences (Chapel Hill, NC, USA). Stock solutions of CUDC-907 and carfilzomib were dissolved in dimethyl sulfoxide. Other reagents were obtained from Sigma-Aldrich (St. Louis, MO, USA).

### Cell lines

The MM cell lines, U266, RPMI8226, MM.1S, MM.1R, and the human bone marrow stromal cell line, HS-5, were obtained from the American Type Culture Collection (ATCC, Manassas, VA, USA). Bortezomib-resistant myeloma cell line, KMS-11/BTZ, was obtained from the Japanese Collection of Research Bioresources Cell Bank (Ibaraki, Osaka, Japan). These MM cell lines were cultured in Roswell Park Memorial Institute (RPMI)-1640 medium containing 10% or 15% fetal bovine serum (FBS) and 1% penicillin/streptomycin and were maintained at 37 °C in a 5% CO_2_ humidified atmosphere. HS-5 cells were cultured in Dulbecco's modified Eagle's medium containing 10% FBS.

### Cell proliferation assays

Cells were treated with CUDC-907 alone or in combination with carfilzomib for 72 h. Viability was evaluated by trypan blue exclusion or with the Cell Counting Kit-8 (Dojindo Laboratories, Mashikimachi, Kumamoto, Japan). This was followed by the measurement of absorbance at 450 nm. All experiments were performed in triplicate. Quantitative determination of synergism or antagonism with the combination index (CI) was performed using the Chou-Talalay method [12].

### Enzyme linked immunosorbent assay (ELISA)

To investigate the Akt activity (Akt is a downstream mediator of PI3K), MM cells were cultured in RPMI medium with or without the indicated concentration of carfilzomib and/or 20% of the volume of HS-5 supernatant. After 48 h, the cells were harvested and stored at − 80 °C. Akt phosphorylation was measured using the Akt Pathway Activation Profile InstantOne™ ELISA Kit (Thermo Fisher Scientific, Waltham, MA). All measurements were performed in triplicate.

### Caspase 3/7 activity

Caspase activity in myeloma cells was examined using the Caspase Glo 3/7 assay kit (Promega, Madison, WI) according to the manufacturer’s instructions. After 48 h of carfilzomib and/or CUDC-907 treatment, the luminescence of each sample was measured using an EnSpire Multimode Plate Reader (PerkinElmer, Waltham, MA).

### Apoptosis assay

Apoptotic cell counts were performed using an Annexin V detection kit (BD Biosciences, Franklin Lakes, NJ) according to the manufacturer’s instructions. Apoptotic cells were analyzed by flow cytometry using an FACS Verse instrument (BD Biosciences).

### Cytotoxicity assay

The cells were treated with various concentrations of CUDC-907 with and without carfilzomib. Cytotoxicity was evaluated based on the release of lactose dehydrogenase (LDH) using the Cytotoxicity LDH Assay kit (Dojindo Laboratories) according to the manufacturer’s instructions. The amount of LDH released from the dead cells was measured using an EnSpire Multimode Plate Reader.

### Histone acetylation assay

The MM cell lines were treated with the indicated concentrations of CUDC-907 for 24 h. After harvesting the cells, cellular histone acetylation was analyzed using the CycLex® Cellular Histone Acetylation Assay Kit (MBL, Nagoya, Aichi, Japan) according to the manufacturer’s instructions. Absorbance was measured for each well using a spectrophotometric reader at the dual wavelengths of 450 and 550 nm.

### HDAC activity assay

The HDAC activity of MM cells was analyzed using the histone deacetylase (HDAC) Activity Assay Kit (Fluorometric) (Abcam, Cambridge, UK) according to the manufacturer’s instructions. Fluorescence intensity signals were measured using a fluorescence microplate reader (Ex/Em 355/460 nm).

### Apoptosis assay

Apoptotic myeloma cells were counted using the Annexin V detection kit (BD Biosciences, Franklin Lakes, NJ, USA) according to the manufacturer’s instructions. Cells were analyzed by flow cytometry using a FACS Verse instrument (BD Biosciences).

### Adenosine triphosphate (ATP) assay

Intracellular ATP levels were determined using the ‘Cell’ ATP assay reagent Ver. two kit (TOYO B-Net, Tokyo, Japan) according to the manufacturer’s instructions. Luminescence was measured using an EnSpire Multimode Plate Reader.

### Proteasome activity

20S proteasome activity was determined using the 20S Proteasome Assay Kit (Cayman Chemical Ann Arbor, MI). The cells were treated with the indicated concentrations of CUDC-907 for 24 h. After harvesting the cells, proteasome activity was analyzed according to the manufacturer’s instructions. Fluorescence intensity was measured using an EnSpire Multimode Plate Reader.

### Analysis of the mitochondrial membrane potential

Mitochondrial membrane potential was analyzed using the cationic JC-1 dye and the Mitochondria Staining Kit (Sigma-Aldrich St. Louis, MO) according to the manufacturer’s instructions. JC-1 monomers and JC-1 aggregates were measured using an EnSpire Multimode Plate Reader.

### Immunoblot analysis

Immunoblot analysis was performed as described previously [13, 14]. After incubation, the cells were washed twice with ice-cold phosphate-buffered saline and lysed using a radioimmunoprecipitation assay lysis buffer. Protein content was quantified using the DC Protein Assay Kit (Bio-Rad, Hercules, CA, USA) according to the manufacturer's instructions. Total cellular proteins (40 µg) were separated on 4%–20% polyacrylamide gels and transferred onto polyvinylidene difluoride membranes. The membranes were probed using appropriate primary antibodies at the appropriate dilutions for 1 h at room temperature. The blots were visualized via chemiluminescence using the Amersham ECL chemiluminescence kit (GE Healthcare, Tokyo, Japan). Specific primary antibodies (Abs) against acetyl histone H4 (Lys5), cleaved caspase 3 (Asp175), poly ADP-ribose polymerase (PARP), phospho-Akt (Ser473), phospho-S6 ribosomal protein (Ser235/236), and NF-κB p65 were purchased from Cell Signaling Technology (Danvers, MA). BCL-XL and MCL-1 were purchased from Santa Cruz Biotechnology (Santa Cruz, CA, USA). Three independent experiments were performed in each case.

### Statistical analysis

All the presented data were analyzed using Prism 9.01 (GraphPad) or Excel software. Student’s t-test was used to determine if the effects on the drug-treated groups were statistically significant compared to those on the control. P < 0.05 or P < 0.01 was considered statistically significant.

## Results

### Effect of proteasome inhibitor in the presence of feeder cells

**Fig. 1:**
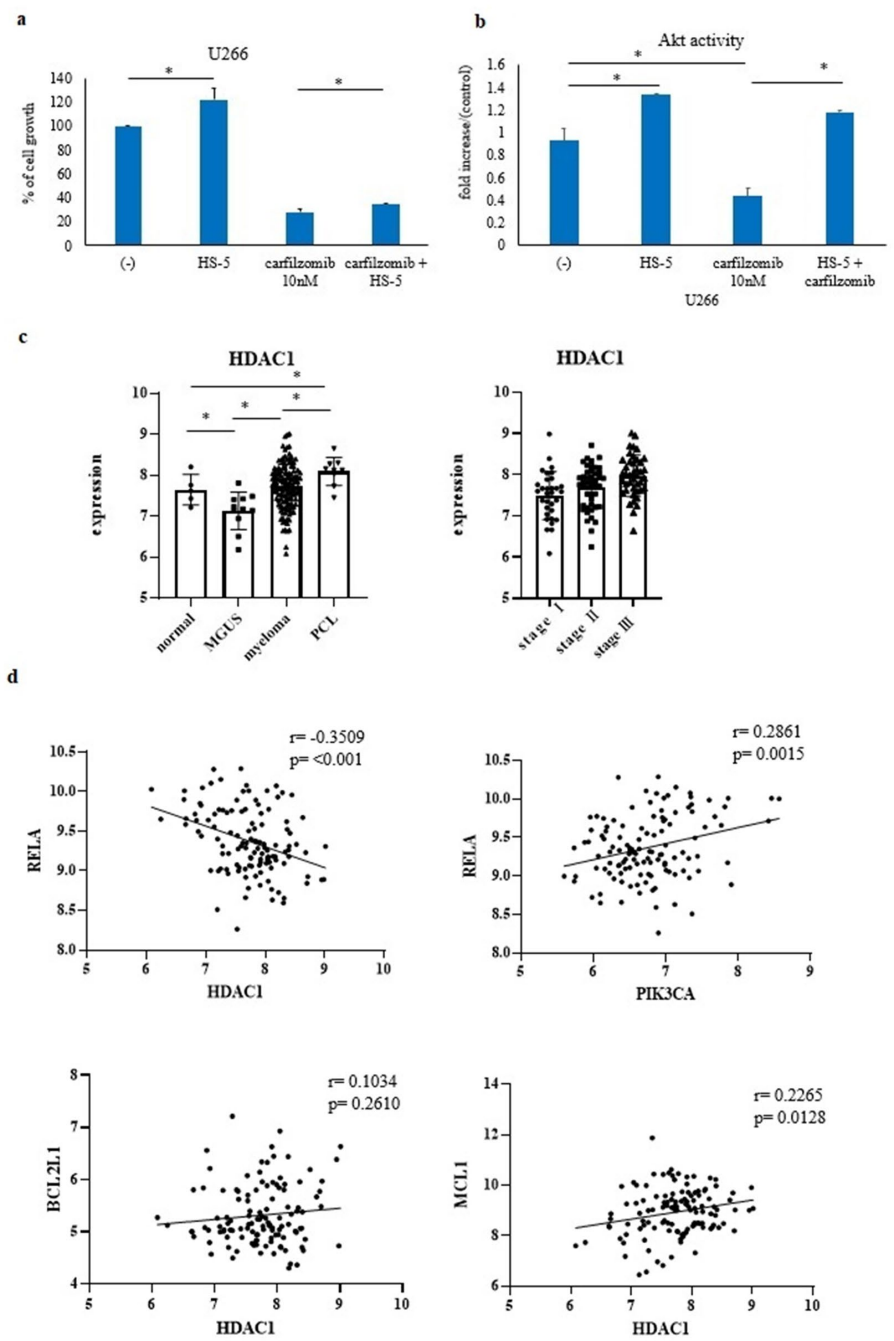
Expression of Akt and HDACs in the myeloma cells. **a**, **b** U266 cells were co-cultured with or without HS-5 cell culture supernatant and treated with carfilzomib (10 nM) for 48 or 72 h. Percentage cell growth (**a**) and Akt activity (**b**) were determined. *P < 0.05 vs. control or HS-5 supernatant treatment group. **c** Gene expression data were analyzed. Validation of HDAC genes by GEO data (GSE13591) in the normal group (n = 5), MGUS group (n = 11), myeloma group (n = 133), and PCL group (n = 9). *P < 0.05, **P < 0.01 vs. control. **d** Correlation between *RELA, BCL2L1*, and *MCL1* with *HDAC1* or *PIK3CA* in the myeloma samples by GEO data (GSE13591). The myeloma group was analyzed.

Proteasome inhibitor activity was analyzed in the presence of the feeder cell line HS-5. We found that co-culture with HS-5 culture supernatant inhibited proteasome activity (Fig. 1a). Moreover, the Akt activity was also analyzed using the AKT Pathway Activation Profile InstantOne™ ELISA Kit. In the presence of the HS-5 supernatant, Akt activity increased even after carfilzomib treatment (Fig. 1b). Thus, PI3K/Akt/ mTOR signaling is activated in the presence of feeder cells.

### Expression of HDAC in myeloma cells

HDACs are essential for transcriptional gene activity [6]. Since class I HDACs play a significant role in the regulation of cell proliferation and cancer, we first investigated the expression of HDAC genes in myeloma cells. The expression of *HDAC* was investigated using Gene Expression Omnibus (GEO) data. *HDAC1* expression reduced in patients with monoclonal gammopathy of undetermined significance (MGUS). However, the number of patients with myeloma and plasma cell leukemia (PCL) was higher than that of patients with MGUS (GSE13591) (Fig. 1c).

### Analysis of correlation between the expression of *HDAC1* and *PIK3CA*

We further investigated the correlation between the expression of *HDAC1* and *PIK3CA*. Based on the GEO data, *BCL2L1*, *MCL1*, and *RELA* genes were analyzed (GSE13591). We found that the expression of *RELA* was positively correlated with *PIK3CA* expression (Fig. 1d) but negatively correlated with *HDAC1* expression (Fig. 1d). The expression of *BCL2L1* and *MCL1* was positively correlated with *HDAC1* expression (Fig. 1d).

### HDAC and Akt activity in myeloma cells

**Fig. 2:**
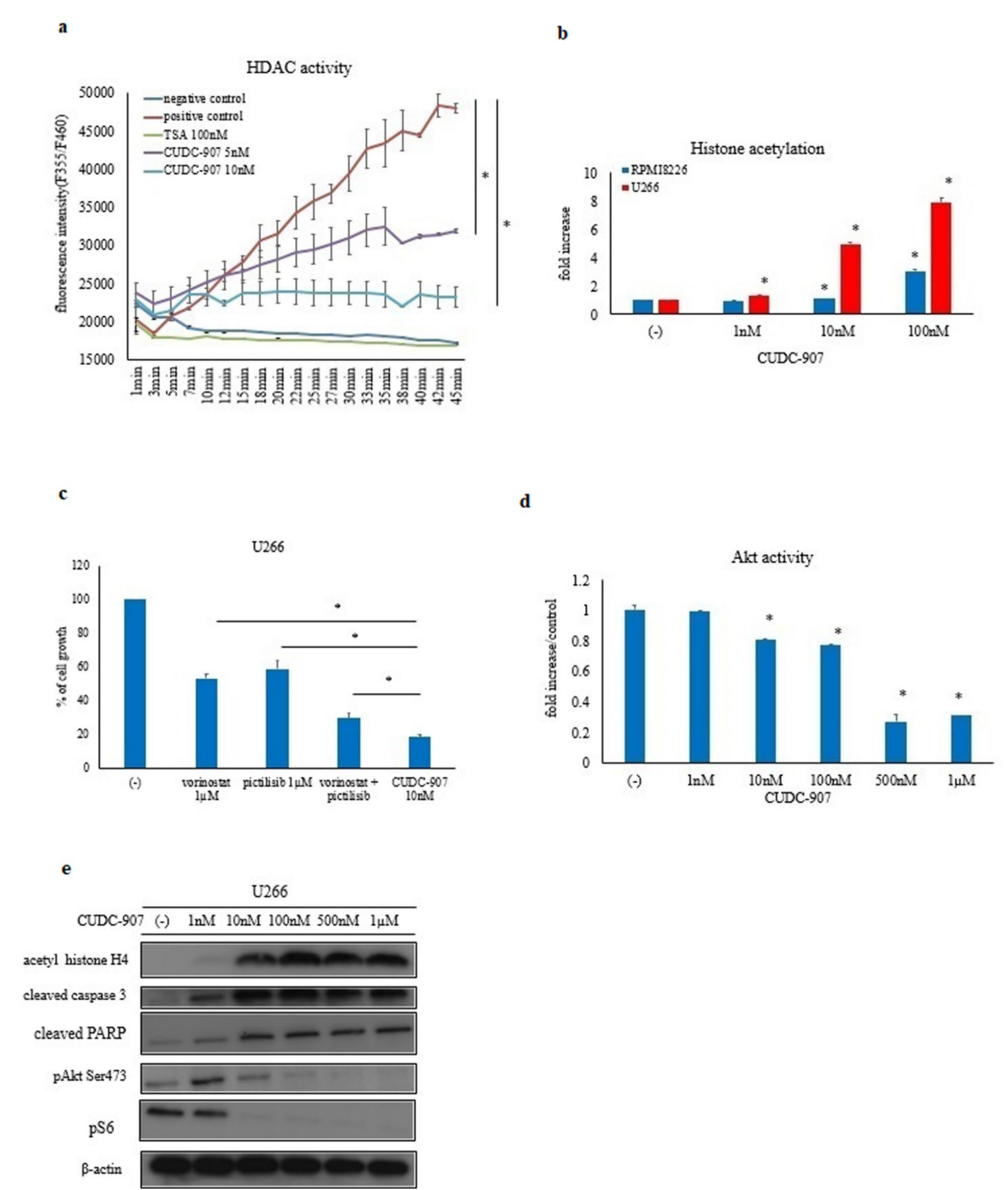
CUDC-907 activity in the myeloma cells. **a** Time course of HDAC activity analyzed using the HDAC Activity Assay Kit. Fluorescence intensity signals were measured using an EnSpire Multimode Plate Reader (Ex/Em 355/460 nm). *P < 0.05 vs. control. **b** Histone acetylation status in CUDC-907-treated (for 24 h) myeloma cells. Cellular histone acetylation was analyzed using the CycLex® Cellular Histone Acetylation Assay Kit. *P < 0.05 vs. control. **c** Myeloma cells were treated with vorinostat, pictilisib, vorinostat plus pictilisib, and CUDC-907 for 72 h. Cell growth was evaluated using the Cell Counting Kit-8. *P < 0.05 vs. CUDC-907-treated group. **d** Cells were treated with the indicated concentrations of CUDC-907 for 24 h. Akt activity was analyzed using the AKT (Phospho) [pS473] Human ELISA Kit. *P < 0.05 vs. control. **e** U266 cells were treated with CUDC-907 for 24 h. Total cell lysates were evaluated by immunoblotting. Results represent the mean of three independent experiments.

Compared to that observed in the GEO data, *HDAC1* expression changed in the myeloma cells. Thus, we investigated HDAC activity in the myeloma cell line U266. The results showed increased HDAC activity in myeloma cells (Fig. 2a). We also found that CUDC-907 inhibited HDAC activity. Treatment with 10 nM CUDC-907 completely inhibited HDAC activity. Acetylation and deacetylation of histones play an important role in transcription regulation. After CUDC-907 treatment, acetylation of histones increased in a dose-dependent manner (Fig. 2b). We then investigated other HDAC and PI3Kα inhibitors, i.e., vorinostat and pictilisib, respectively. Co-treatment with vorinostat and pictilisib induced cell growth inhibition in myeloma cells (Fig. 2c). It was found that the cell growth inhibition by 10 nM of CUDC-907 was better than that by 1 µM vorinostat and 1 µM pictilisib. Akt is a protein kinase that can be activated by various growth factors in the PI3 kinase pathway. In this study, we investigated Akt activity; Akt is a downstream mediator of PI3K [9]. CUDC-907 reduced Akt activity in a dose-dependent manner (Fig. 2d). Immunoblot analysis revealed that the levels of acetyl histone H4, cleaved caspase 3, and cleaved PARP increased, whereas phosphorylation of Akt and downstream molecule S6 ribosomal protein decreased upon CUDC-907 treatment (Fig. 2e).

### Effect of CUDC-907 on myeloma cells

**Fig. 3:**
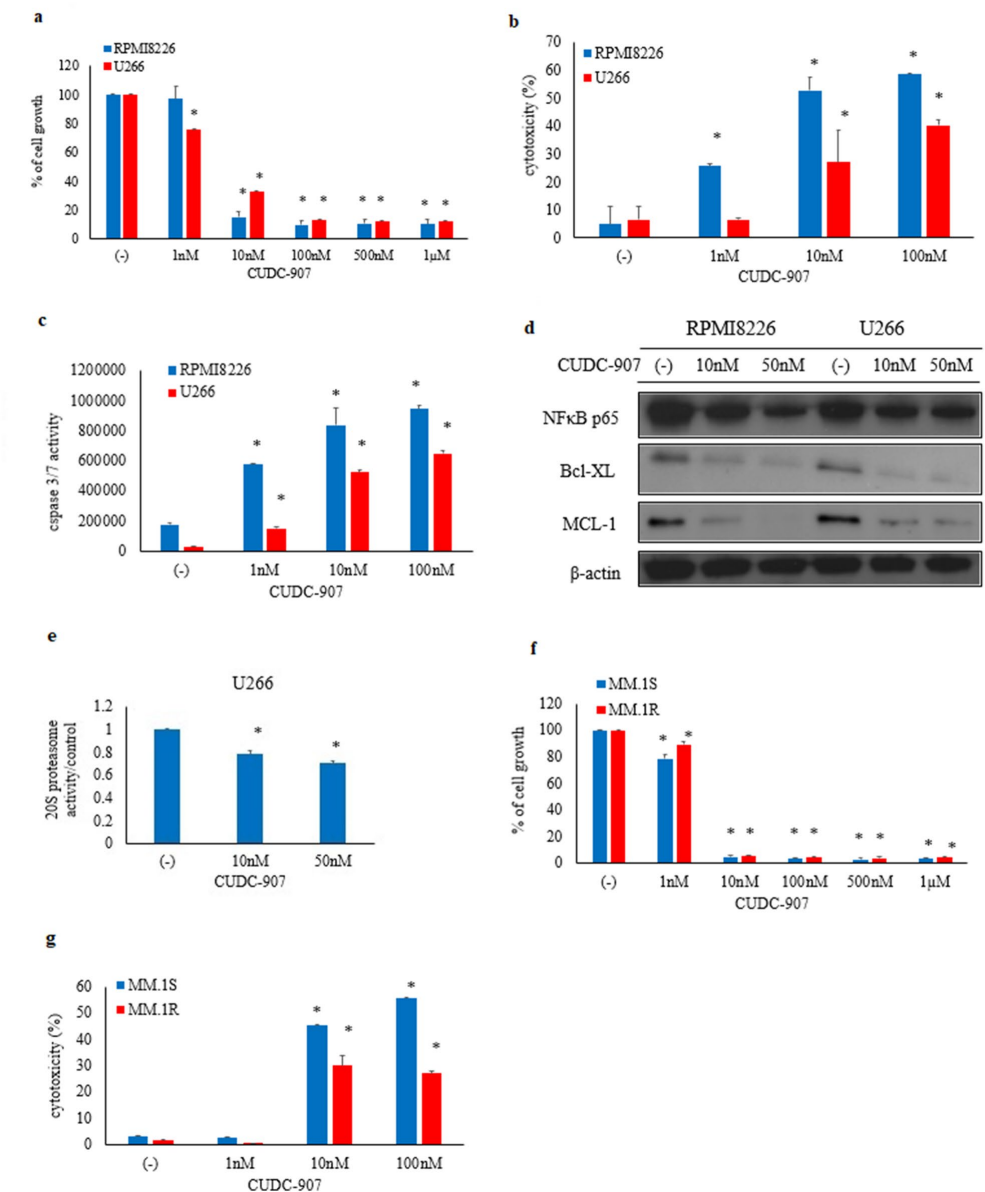
Efficacy of CUDC-907 in the myeloma cells. Myeloma cells were treated with the indicated concentrations of CUDC-907 for 48 h or 72 h. The effect of CUDC-907 treatment on cellular growth (**a**, **f**), cytotoxicity (**b**, **g**), and caspase 3/7 activity (**c**) was evaluated. *P < 0.05 vs. control. **d** Myeloma cells were treated with the indicated concentration of CUDC-907 for 24 h. Total cell lysates were evaluated by immunoblotting. Results represent the mean of three independent experiments. **e** Myeloma cells were treated with CUDC-907 for 24 h and measured using the 20S Proteasome Assay Kit. *P < 0.05 vs. control

CUDC-907 potently inhibits class I PI3Ks as well as class I and II HDAC enzymes [15]. We further examined the efficacy of CUDC-907 against MM cells. The MM cell lines were incubated with the indicated concentrations of CUDC-907 to analyze its anti-proliferative effect. Treatment with CUDC-907 for 72 h induced cytotoxicity in the MM cell lines RPMI8226 and U266 in a dose-dependent manner (Fig. 3a). LDH assay was performed as another indicator of myeloma cell cytotoxicity, and it showed an increase in cellular cytotoxicity after CUDC-907 treatment (Fig. 3b). The caspase 3/7 activity in myeloma cells was then investigated. An elevation in caspase 3/7 activity levels upon CUDC-907 treatment was observed (Fig. 3c). Immunoblot analysis revealed that the protein expression of NF-κB p65 (RelA), BCL-XL, and MCL-1 was reduced upon CUDC-907 treatment (Fig. 3d). Since the expression of NF-κB p65 (RelA) was reduced upon CUDC-907 treatment, we investigated the proteasome activity. We found that the 20S proteasome activity was reduced upon CUDC-907 treatment in a dose-dependent manner (Fig. 3e). We also found CUDC-907 induced cytotoxicity in the other MM cell lines (MM.1S and MM.1R) in a dose-dependent manner (Fig. 3f, 3g).

### CUDC-907 enhances the activity of the proteasome inhibitor in myeloma cell lines

**Fig. 4:**
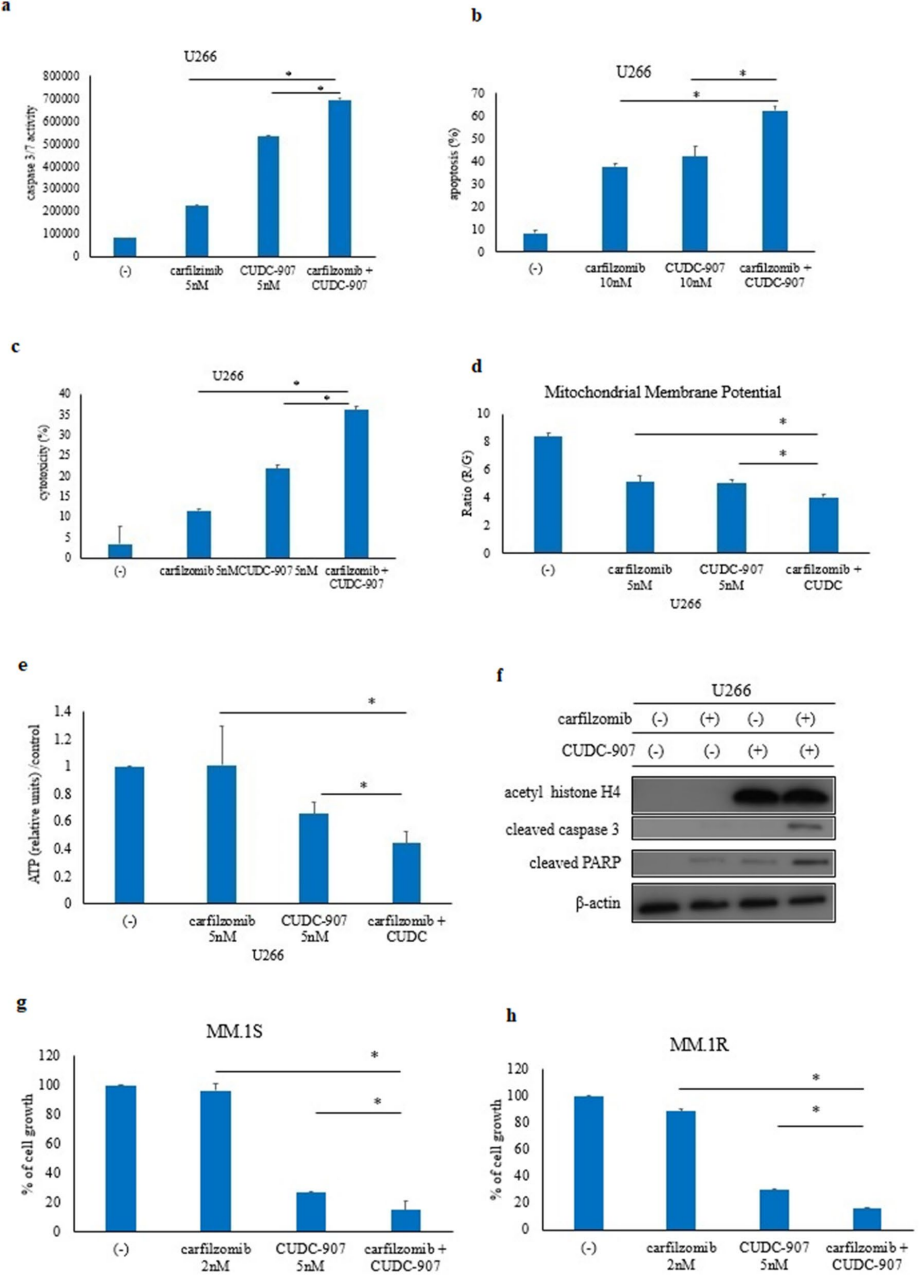
Co-treatment with carfilzomib and CUDC-907 induces cytotoxicity in the myeloma cells. **a**–**c** The myeloma cells were treated with carfilzomib and/or CUDC-907 for 48 h or 72 h. Caspase 3/7 activity (**a**), apoptotic cells (**b**), and cytotoxicity (**c**) were analyzed. ^*^P < 0.05 compared to carfilzomib and CUDC-907 co-treated cells. **d** Myeloma cells were treated with carfilzomib and/or CUDC-907 for 48 h. Mitochondrial membrane potential was determined using the Mitochondria Staining Kit. ^*^P < 0.05 compared to carfilzomib and CUDC-907 co-treated cells. **e** Cells were incubated with carfilzomib and/or CUDC-907 for 72 h. Intracellular ATP levels were determined using the ‘Cell’ ATP assay reagent Ver. 2 kit. *P < 0.05 vs. carfilzomib and CUDC-907 co-treatment groups. **f** Myeloma cells were treated with the indicated concentrations of carfilzomib and/or CUDC-907 for 24 h. Total cell lysates were evaluated by immunoblotting. **g**, **h** Myeloma cells were treated with the indicated concentrations of CUDC-907 for 72 h. The effect of CUDC-907 treatment on cellular growth (**g**) and cytotoxicity (**h**) was evaluated. ^*^P < 0.05 compared to carfilzomib and CUDC-907 co-treated cells. Results represent the mean of three independent experiments

We investigated whether carfilzomib, a proteasome inhibitor, and CUDC-907 enhanced cell growth inhibition in myeloma cells. The myeloma cells were cultured with carfilzomib and/or CUDC-907 for 72 h, harvested, and then, the cell proliferation assay was performed. The growth of myeloma cells was reduced more efficiently by the combination of carfilzomib and CUDC-907 when compared with the independent application of each drug (Additional file 1: Fig. S1a, b). The results of our drug combination studies were further analyzed using the Chou-Talalay method for synergy quantification. The CI values were found to be less than 1, indicating that these drug combinations were synergistic (data not shown). Caspase 3/7 activity was increased after carfilzomib and CUDC-907 co-treatment when compared with the independent application of each drug (Fig. 4a). Apoptotic cells also increased in number after carfilzomib and CUDC-907 co-treatment (Fig. 4b). Cellular cytotoxicity was also enhanced (Fig. 4c). Mitochondrial membrane potential is a key indicator of mitochondrial activity and is an essential component in the process of energy storage during oxidative phosphorylation [16]; therefore, we used a Mitochondria Staining Kit. Mitochondrial uptake of JC-1 may be used as an effective distinction between apoptotic and healthy cells. The relative disrupted mitochondrial ratio was increased in carfilzomib and CUDC-907 co-treatment (Fig. 4d). ATP levels were reduced upon carfilzomib and CUDC-907 co-treatment (Fig. 4e). In the immunoblot analysis, acetyl histone H4 was increased in the presence of CUDC-907. Cleaved caspase 3 and cleaved PARP were enhanced after carfilzomib and CUDC-907 co-treatment (Fig. 4f). We also found that the growth of myeloma cells was reduced more efficiently by carfilzomib and CUDC-907 in the MM.1S and MM.1R cell lines (Fig. 4g, h). These results indicated that co-treatment with carfilzomib and CUDC-907 enhanced cellular toxicity in myeloma cells.

### CUDC-907 treatment inhibited proteasome inhibitor-resistant myeloma growth

**Fig. 5:**
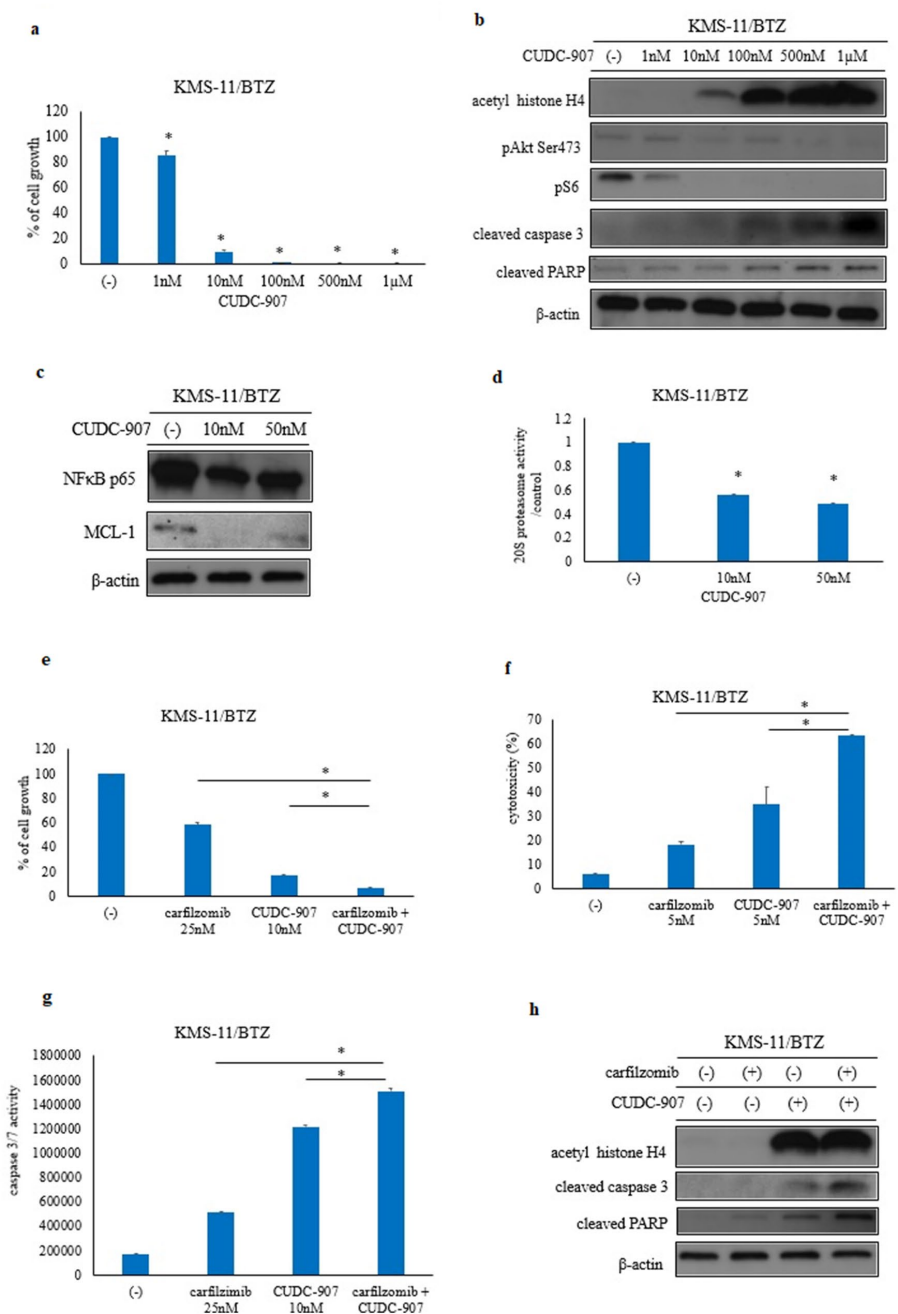
Efficacy of CUDC-907 in the proteasome inhibitor-resistant myeloma cells. **a** KMS-11/BTZ cells were treated with the indicated concentrations of CUDC-907 for 72 h. The effect of CUDC-907 treatment on cellular growth was evaluated. *P < 0.05 vs. control. **b** KMS-11/BTZ cells were treated with CUDC-907 for 24 h. Total cell lysates were evaluated by immunoblotting. Results represent the mean of three independent experiments. **c** Myeloma cells were treated with the indicated concentration of CUDC-907 for 24 h. Total cell lysates were evaluated by immunoblotting. **d** Myeloma cells were treated with CUDC-907 for 24 h and measured using the 20S Proteasome Assay Kit. *P < 0.05 vs. control. **e**, **f**, **g** The effect of CUDC-907 treatment on cellular growth (**e**) and cytotoxicity (**f**) and caspase 3/7 activity (**g**) was evaluated. ^*^P < 0.05 compared to carfilzomib and CUDC-907 co-treated cells. **h** Myeloma cells were treated with the indicated concentrations of carfilzomib and/or CUDC-907 for 24 h. Total cell lysates were evaluated by immunoblotting.

Bortezomib-resistant KMS-11/BTZ cells were treated for 72 h with CUDC-907. CUDC-907 induced cytotoxicity in KMS-11/BTZ in a dose-dependent manner (Fig. 5a). Immunoblot analysis revealed that the levels of acetyl histone H4, cleaved caspase 3, and cleaved PARP were increased. In addition, phosphorylation of Akt and downstream molecule S6 ribosomal protein was decreased (Fig. 5b). The protein expression of NF-κB p65 (RelA) and MCL-1 was reduced by CUDC-907 (Fig. 5c). 20S proteasome activity was also reduced upon CUDC-907 treatment in a dose-dependent manner (Fig. 5d). The proliferation of KMS-11/BTZ cells was reduced more efficiently by the combination of carfilzomib and CUDC-907 when compared with the independent application of each drug (Fig. 5e–g). In the immunoblot analysis, acetyl histone H4 was increased in the presence of CUDC-907. In addition, cleaved caspase 3 and cleaved PARP were enhanced after carfilzomib and CUDC-907 co-treatment (Fig. 5h). These results indicated that co-treatment with carfilzomib and CUDC-907 enhanced cellular toxicity in bortezomib-resistant myeloma cells.

## Discussion

The PI3K/Akt-signaling pathway is one of the most frequently activated signal transduction pathways in cancers [9]. Dysregulated HDAC activity, which is regulated by epigenetic gene expression, was also identified in cancer cells [7]. In this study, we demonstrated that the PI3K/Akt pathway was activated in the presence of feeder cells (Fig. 1b). Moreover, we confirmed that the expression of class 1 HDAC changed in myeloma cells (Fig. 1c), indicating that the dual targeting of the PI3K/Akt pathway and HDAC may become an important strategy for the treatment of myeloma.

In this study, we investigated the effect of CUDC-907 on human myeloma cells in vitro. Although single-target inhibitors of either the PI3K/Akt/mTOR pathway or HDACs would be good drug candidates for cancer therapy, their demonstrated efficacies are limited due to their unfavorable pharmaceutical activities, toxicity, and crossover inhibition of other lipid and protein kinases [17]. CUDC-907 is a dual inhibitor of PI3K and HDAC for PI3Kα and HDAC1/2/3/10, respectively. Its use led to sustained inhibition of HDAC and PI3K activity. Our results showed that CUDC-907 potently inhibited the proliferation of myeloma cell lines at a lower concentration (Fig. 3a) by inhibiting Akt, which is a downstream mediator of PI3K (Fig. 2d). Moreover, CUDC-907 was more efficacious than either PI3K or HDAC inhibitors alone (Fig. 2c).

Our results showed that CUDC-907 downregulated the expression of cell survival proteins such as RELA, BCL-XL, and MCL-1 (Fig. 3D). RELA is the nuclear factor NF-κB p65 subunit in humans [18]. Since the constitutive upregulation of NF-κB activity is associated with chemoresistance [19], inhibition of NF-κB may increase the susceptibility to anti-myeloma treatment. We confirmed that 20S proteasomal activity was reduced by CUDC-907. BCL-XL is also an anti-apoptotic molecule known to play a role in development and differentiation [20]. Immunoblot analysis revealed that the expression of BCL-XL and MCL-1 was reduced, suggesting the CUDC-907-induced death of myeloma cells in vitro.

CUDC-907 is currently under clinical trial with regard to hematological malignancies and solid tumors. Younes et al. have reported that CUDC-907 is safety and activity in this dose-escalation study of CUDC-907 monotherapy in patients with relapsed or refractory lymphoma [21]. Oki et al. have presented the safety and preliminary activity results of CUDC-907, with and without rituximab, in patients with relapsed/refractory diffuse large B-cell lymphoma (DLBCL), which is expressed with MYC-altered disease [22]. Li et al. have reported that CUDC-907 treatment decreases the number of leukemia progenitor cells in primary acute myeloid leukemia (AML) [23]. It has also been reported that the activity of CUDC-907 in ABCG2-overexpressing cancer cells can be restored by inhibiting the function of the ATP-binding cassette drug transporter, ABCG2, which is one of the most common mechanisms for multidrug resistance in cancers [24].

## Conclusion

In conclusion, CUDC-907 inhibits the proliferation of myeloma cells. CUDC-907 increases the activity of carfilzomib, a proteasome inhibitor, in vitro. The use of combination therapy focused on carfilzomib and CUDC-907 may be a potentially good therapeutic strategy to treat the relapse of/refractory myeloma.

## Supplementary Information

**Additional file 1: Figure S1.** Efficacy of carfilzomib and CUDC-907 in myeloma cells. RPMI8226 (**a**) and U266 (**b**) cells were treated with carfilzomib and/or CUDC-907 for 72 h. The relative cell growth rates were determined.

## Abbreviations

MM: Multiple myeloma
HDAC: Histone deacetylase
PI3K: Phosphoinositide 3-kinase
mTOR: Mammalian target of rapamycin
FBS: Fetal bovine serum
LDH: Lactose dehydrogenase
CI: Combination index
ELISA: Enzyme-linked immunosorbent assay
ATP: Adenosine triphosphate
Abs: Antibodies
PARP: Poly ADP-ribose polymerase
GEO: Gene Expression Omnibus
MGUS: Monoclonal gammopathy of undetermined significance
PCL: Plasma cell leukemia
DLBCL: Diffuse large B-cell lymphoma
AML: Acute myeloid leukemia
