# Supplementary figures and images for: Targeting phosphoinositide 3-kinases and histone deacetylases in multiple myeloma
Source: Exp Hematol Oncol. 2021 Mar 4;10:19. doi: 10.1186/s40164-021-00213-6 (PMC7934550; doi:10.1186/s40164-021-00213-6)

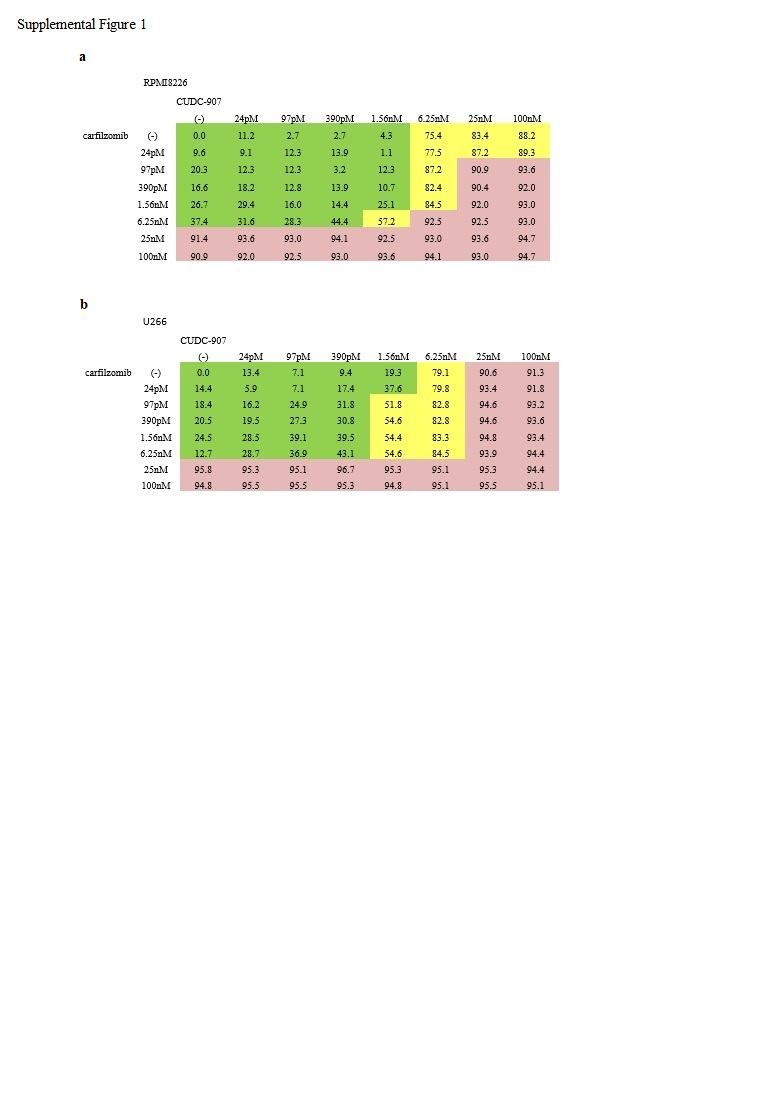

Supplement: Supplementary file 1 — Additional file 1: Figure S1. Efficacy of carfilzomib and CUDC-907 in myeloma cells. RPMI8226 (a) and U266 (b) cells were treated with carfilzomib and/or CUDC-907 for 72 h. The relative cell growth rates were determined. [file 40164_2021_213_MOESM1_ESM.tif]
